# Effects of Imagined Consumption and Simulated Eating Movements on Food Intake: Thoughts about Food Are Not Always of Advantage

**DOI:** 10.3389/fpsyg.2016.01691

**Published:** 2016-10-28

**Authors:** Simona Haasova, Botond Elekes, Benjamin Missbach, Arnd Florack

**Affiliations:** ^1^Department of Psychology, University of ViennaVienna, Austria; ^2^Department of Nutritional Sciences, University of ViennaVienna, Austria

**Keywords:** mental imagery, habituation, food intake regulation, motor simulation, eating

## Abstract

Imagined food consumption is a method of elaborately imagining oneself eating a specific food that, when repeated 30 times, has been shown to decrease subsequent intake of the same food. The technique relies on a memory-based habituation process when behavioral and motivational responses to a stimulus decrease after its repeated presentation. Thus, repeatedly imagining food consumption leads to food-specific habituation effects. Large numbers of imagined consumption repetitions are effortful and time consuming and can be problematic when applied in interventions with the goal of reducing food intake. In the present study, we assessed the efficacy of the technique at smaller numbers of repetitions while testing motor simulation as a potential facilitator of the habituation-based consumption-reduction effect. 147 participants imagined eating chocolate pudding 15 or 3 consecutive times and simultaneously performed either facilitating or not-facilitating eating movements. Results showed that participants who imagined eating the chocolate pudding 15 times (*M*_15_ = 178.20, *SD*_15_ = 68.08) ate more of the pudding than those who imagined consuming it 3 times (*M*_3_ = 150.73, *SD*_3_ = 73.31). The nature of the motor movements that were performed did not impact this effect. The data suggest that the imagined food consumption technique can result in an unexpected increase in food consumption, when smaller numbers of imagination repetitions are performed.

## Introduction

It is a common experience that merely thinking about eating delicious food makes the mouth water and bolsters the desire to eat it. Seeing, smelling or even thinking about favorite food represents a tempting cue that induces subjective feelings of craving (for a review see: Petrovich, 2013; Boswell and Kober, 2016) as well as preparatory physiological responses such as an increase in hunger (e.g., Jansen and van den Hout, 1991; Staiger et al., 2000) and salivation (Nederkoorn and Jansen, 2002). As a result, higher food consumption follows that can further motivate overeating and weight gain (e.g., Jansen et al., 2011; Rodríguez-Martín and Meule, 2015). This may also be true in cases when one does not feel any hunger (Pelchat and Schaefer, 2000).

One would typically assume that the desire to eat becomes stronger the more one thinks about it. And indeed, research on inhibitory control in the area of eating behavior suggests that such intrusive thoughts ought to be deliberately suppressed (Anderson and Bjork, 1994), inhibiting retrieval of food-related information from memory (Davidson et al., 2005) in order to prevent consumption in situations when it is undesirable. Interestingly, recent research found that multiple repetitions of these “consumption” thoughts not only halt further increases in the desire to eat the food but also decrease the desire and reduce actual consumption of a specific food that participants imagined themselves eating (Morewedge et al., 2010; Missbach et al., 2014). This “paradoxical” decrease of consumption desire after its repeated rehearsal has been suggested to reflect habituation effects (Morewedge et al., 2010). The habituation mechanism is defined by decreases in biological, motivational, and behavioral responses to food due to its repeated presentation (Epstein et al., 2009). Only after repeated presentations, when the food stimulus matches the previously stored information in memory, does the processing of the food stimulus, along with the responsiveness to it, decrease (Epstein et al., 2009). Research has demonstrated habituation effects across different types of responses such as salivation, motivated responding, and food intake, and with different food types such as cheeseburgers, cheese, M&Ms, gummy bears, and walnuts (Epstein et al., 2003; Morewedge et al., 2010; Missbach et al., 2014). Habituation was also found across different types of paradigms such as repeated exposure to actual food stimuli (e.g., Epstein et al., 2003, 2009) and repeated imagined consumption of a particular food (Morewedge et al., 2010; Missbach et al., 2014). Studies showed that imagining eating M&Ms 30 times resulted in a smaller amount of subsequently eaten M&Ms than when participants imagined eating them only three times (Morewedge et al., 2010).

In light of these intriguing findings, the next step is to utilize the repeated imagined-consumption paradigm as an intervention that can be applied to decrease the consumption of specific food types (Missbach et al., 2015). Unlike memory inhibition skills that are useful from preventing cues in the environment to retrieve thoughts about eating desires (Davidson et al., 2005), the technique of imagined consumption might potentially become a weight management tool that could be applied as an effective strategy when already being confronted with a cue-elicited eating desire (e.g., Jansen et al., 2003). For example, similarly to the popular strategy of counting till 10 to calm down when facing a stressful situation, in the face of a temptation, one might first imagine eating the tempting food 10 times before deciding whether to indulge. The imagined consumption technique has a potential to be applied as a stand-alone and spot-on intervention to curb eating behavior or as a part of a more complex behavior-change technique, for example, as part of an implementation intention (Gollwitzer, 1999). The appeal of such action planning techniques lies mainly in their parsimony, low costs and low response burden that allows them to be applied easily and effectively (Hagger and Luszczynska, 2014). Therefore, there is first a need to establish the most effective application conditions by identifying factors that can enhance the strength of the imagined consumption technique.

This need is further corroborated by the fact that the application of this paradigm might be rendered difficult when using larger numbers of imagined consumption repetitions. Morewedge et al. (2010), for instance, asked participants to repeat the imagination of consumption 33 or 30 times. Engaging in such a large number of repetitions requires more time and a very high level of motivation and self-control and it is conceivable that the application difficulty of the task might cause reduced compliance and thus threaten the goal of the intervention. Indeed, previous research showed that the availability of self-regulatory resources is necessary for the reduction in food intake induced by repeated imagined consumption to occur (Missbach et al., 2014, Study 2).

The objective of the present research was to continue examining the efficacy of the repeated imagined-consumption paradigm by using a feasible number of repetitions that can be included in interventions. While most studies apply more than 30 repetitions, there are hints that imagined-consumption paradigm can be effective at lower number of repetitions. For example, the mentioned study of Missbach et al. (2014, Study 2) successfully applied 18 repetitions. However, the same study showed that the effect at this number of repetitions has limits, and does not occur for depleted participants. Indeed, it is highly likely that at lower numbers of repetition sensitization effects can occur that either reduce habituation or increase the food intake. Therefore, we tested whether motor simulation could further enhance the consumption-reduction effect of repeatedly imagining consumption at a lower number of repetitions.

Motor movements represent an inseparable part of consumption and recent research by Elder and Krishna (2012) suggests that the visualization of consumption involves a complex simulation pattern that can be supported by the facilitation of motor movements. In their study, presenting food images and visually facilitating the motor movements associated with the food led to greater visualization of food consumption. Moreover, triggering movements related to approaching food (Förster, 2003) and mouth movements (Topolinski and Boecker, 2016) increased consumption and food attractiveness, respectively. Grounded cognition theory posits that presentation of a particular stimulus, food for example, activates sensory perceptions and mental simulation of prior attained representation of and interaction with that object, such as an act of eating the food (Barsalou, 2008). Because perception and mental simulation overlap greatly in their use of cognitive resources and brain-area activation (Ganis et al., 2004), and mental simulation of an interaction with an object can activate the same sensory-motor regions of the brain associated with actual object-interaction (Barsalou, 2008), we propose that food-associated motor movements might boost the consumption-reduction effect at lower repetitions of imagined consumption. Rehearsal of motor movements that facilitate consumption experience might enhance the short-term memory by helping to recall and prime the food information during imagined consumption. This might accelerate the assumingly underlying habituation, which is a memory-based process occurring when the (imagined) food stimulus matches the information previously stored in memory (Epstein et al., 2009).

In the present research, we disguised the study as a taste test of a chocolate pudding. The alleged taste test was preceded by the imagined-consumption paradigm in combination with a motor movement simulation task. The paradigm consisted of a mental imagery task, based on similar tasks used by Missbach et al. (2014) and Morewedge et al. (2010) and involved repeatedly imagining oneself consuming the chocolate pudding. We employed 15 and 3 repetitions of imagining the consumption and a simulation of eating-associated motor movements. We hypothesized that the habituation, leading to a decrease in actual food consumption, would occur after 15 in comparison with 3 repetitions. We further assumed that facilitating (vs. not-facilitating) motor movements during repeatedly imagining consumption would strengthen habituation, resulting in an amplified reduction of food intake. Nevertheless, even though previous research was successful at showing habituation effects after 18 repetitions of imagined consumption (Missbach et al., 2014), habituation effects at 15 repetitions have not been previously examined. Therefore, there is a possibility that at the lower amount of 15 imagination repetitions the habituation will fully develop and manifest itself as significant decrease of food intake only when facilitated by simulated eating movements.

## Methods

### Participants

One hundred fifty-nine students from the University of Vienna volunteered to participate in the laboratory experiment in exchange for study credits or the opportunity to win 5 EUR in a lottery. We recruited participants via social media and flyers posted on the University campus. Participants were asked to refrain from eating and drinking soft drinks 2 h prior to the experiment and believed they were taking part in a taste test. We excluded 12 participants from the data analysis due to either an initially reported dislike toward eating the food—chocolate pudding—in the study or no response on the “liking” measure (8), due to expressed aversion to eat the food during the “taste test” task (2), due to incomplete responses on the online questionnaires containing measures of control variables (1) and because one participant explained to eat less in the “taste test” task than desired, being afraid to experience allergic reaction. The final sample consisted of 147 participants (115 female) with a mean age of 24.37 years (*SD* = 5.14).

### Design and procedure

Participants were randomly assigned to a 2 (imagined consumption repetitions: 15 vs. 3) × 2 (motor simulation: facilitating vs. not-facilitating) between-subjects design. Prior to the experiment, participants completed an online questionnaire on restrained eating, subjective dieting success, and additional questions on eating self-regulation. When arriving at the laboratory, participants signed the informed consent and answered questions about their age, gender, height, and weight and indicated their current hunger, mood, liking of chocolate pudding and their weekly frequency of sweets consumption. The alleged taste test was preceded by the mental imagery task, when participants repeatedly imagined consuming pudding either 15 or 3 consecutive times. To facilitate (vs. not facilitate) the motor aspect of consumption, participants concurrently performed hand and mouth movements that are typically associated with eating chocolate pudding (vs. holding the tongue behind the teeth).

During the joint task, participants listened to audio recordings where they were instructed by a calm, female voice to imagine how they would eat the chocolate pudding. They were told to repeat imagining the consumption 3 or 15 times, receiving instructions with each of the ascribed repetitions, holding the time for each imagination constant at about 40 s. We instructed the participants to imagine how they put a spoon into the chocolate pudding, move the spoon toward their mouth, smell the scent of the pudding, taste and swallow it, emphasizing the visual, olfactory and haptic properties of the food. Simultaneously, along with each consumption imagination, participants were instructed to actually move their hand holding an imagined spoon to the mouth, open it and swallow (consumption facilitating movement), or to keep their hands still and hold their tongue behind their teeth (consumption not-facilitating movements), respective of their assigned condition. As a manipulation check, we asked participants to draw a dash on a sheet of paper every time they successfully managed to imagine the consumption. Next, participants reported on subjectively perceived cognitive demand during the imagery and motor simulation task, and subjective quality of imaginations. Afterwards, participants engaged in an 8 min long taste test of the previously imagined chocolate pudding during which they could eat *ad libitum* from a bowl containing 250 g of the pudding. We removed the bowl when participants indicated they were finished and measured the amount an individual had consumed, otherwise not being present during participants' consumption. Subsequently, following the taste test scenario, participants answered evaluative questions about the chocolate pudding (e.g., “What did you like best about the chocolate pudding?”) and again reported their current hunger, mood and liking of the chocolate pudding (for more details, see Supplementary Datasheet 1).

### Measures

We have collected measures of participant‘s hunger and liking of the chocolate pudding in order to control for individual motivation to eat. For this reason, we asked participants to indicate their current mood, because previous research showed that negative mood can increase consumption of hedonic foods (e.g., Garg et al., 2007). Further, we have also included questionnaires on individual eating, dietary and regulatory characteristics. Restrained eaters were previously found to eat significantly more after exposure to food cues than unrestrained eaters (Fedoroff et al., 1997) and self-perceived success in dieting correlates for example with food cravings and binge eating (Meule et al., 2012). We assessed restrained eating with the Restrained eating subscale [10 items on a 5-point rating scale ranging from 1 (*never*) to 5 (*very often*)] from the Dutch Eating Behavior Questionnaire, using its established German version (Van Strien et al., 1986; Grunert, 1989). Dieting success was measured by the Perceived Self-Regulatory Success in Dieting Scale (Meule et al., 2012; three items on a 7-point rating scale ranging from 1 (*not at all difficult/not at all good*) to 7 (*very difficult/very good*). Eating regulation was further assessed by four items from The Advanced Self Regulatory Scale [developed in our laboratory; 7-point rating scale ranging from 1 (*I don't agree at all*) to 7 (*I fully agree*)]. The four items were: “When I am full, I stop eating.”; “It is easy for me to stop eating, when I feel no more hunger.”; “I often eat further, even though my stomach feels full.”; “I often keep eating, even though I am not hungry anymore.” Moreover, we assessed individual frequency of sweets consumption and BMI, because previous research indicated that obese in comparison to non-obese participants can exhibit slower rates of habituation (Temple et al., 2007). Individuals' BMI was calculated as weight (in kilogram) divided by the squared size of height (in meters). Because it is possible that imagining consumption 15 times in comparison to 3 might lead to varying experience of cognitive demand during the experimental task that can in turn influence food intake (e.g., Ward and Mann, 2000), we also asked the participants “How demanding did you perceive the task altogether?” [400 point visual analog scale “VAS,” ranging from 1 (*not at all demanding*) to 400 (*very demanding*)]. Furthermore, to address the role of quality of the consumption imaginations, we asked the participants: “How well could you imagine consuming the food?” [400 point VAS ranging from 1 (*very badly*) to 400 (*very good*)]. How well and vividly can one imagine to consume a particular food was previously suggested by Missbach et al. (2014) to be a potential precondition for successful occurrence of habituation. Lastly, in order to rule out effects of demand characteristics, we also assessed participants' expectations of consumption increase or decrease as a function of number of consumption imaginations and type of motor movement simulation. We used two items: 1. “According to your opinion, how does imagining eating a particular food influence the subsequent consumption of the same food?”(4-point rating scale: 1 = *One eats less than when no imagination takes place.*, 2 = *One eats similar than when no imagination takes place*., 3 = *One eats more than when no imagination takes place*., 4 = *Other*.), 2. “According to your opinion, how does a (non) facilitation of eating movements during consumption imagination influence the subsequent food intake of the same food?” (4-point rating scale: 1 = *Facilitating movements increase the consumed amount, while the not-facilitating movements decrease the consumed amount of food.*, 2 = *There is no influence on the consumed amount.*, 3 = *Facilitating movements decrease the consumed amount, while the not-facilitating movements increase the consumed amount.*, 4 = *Other*.).

### Ethics statement

According to the Austrian Universities Act 2002 UG2002 (Universities Act (UG) BGBl. I No. 120/2002), which was in place at the time the study was carried out, only medical universities were required to appoint ethics committees for clinical tests, application of medical methods, and applied medical research. Consequently, no ethical approval for this specific study was required. Nevertheless, the present study was conducted in accordance with the Declaration of Helsinki (revised 1983) and local guidelines of the Faculty of Psychology, University of Vienna. Written informed consent was given by all participants, who could also withdraw at any time during the experiment without further consequences. At the end of the experiment, participants were debriefed in detail.

### Data analysis

To test our hypotheses, we first performed a between-subjects ANOVA with imagined consumption repetitions (15 vs. 3) and type of motor simulation (facilitating vs. not-facilitating) as independent variables and amount of consumed pudding as the dependent variable. To assess whether cognitive demand, quality of imaginations and change in perceived hunger, mood or liking of the chocolate pudding was influenced by the experimental manipulation and could be considered a control or mediating variable, separate ANOVAs were conducted. Subsequently, we performed an ANCOVA on the consumption scores while controlling for cognitive demand and imaginations quality. Results were considered significant at an α level of *p* ≤ 0.05.

## Results

Descriptives for the four experimental conditions are presented in Table 1. The four experimental conditions did not differ in participants' restrained eating, perceived dieting success, eating self-regulation, BMI or age. At the beginning of the experimental task, participants reported similar feelings of hunger, mood, chocolate pudding liking, and similar frequency of sweets consumption in all experimental conditions. Gender distribution also did not differ between the groups, χ^2^ (3, *N* = 147) = 6.18, *p* = 0.10. All participants indicated they have fully complied with the given task instructions and reported that in the 15 repetition condition they were successful in imagining the consumption of the chocolate pudding on average 10.49 times (*SD*_15_ = 3.83), while in the 3 repetition condition they reported to successfully manage it on average 2.58 times (*SD*_3_ = 0.76), *F*_(1, 143)_ = 299.36, *p* < 0.001, $\eta_{p}^{2}$ = 0.68.

**Table 1:** **Descriptive (means and standard deviations) statistics of variables as a function of the experimental conditions and inferential statistics for group comparisons**.

**Variables**	**Experimental conditions**					
	**3 repetitions motor facilitation (*N* = 37)**	**3 repetitions motor not-facilitation (*N* = 37)**	**15 repetitions motor facilitation (*N* = 38)**	**15 repetitions motor not-facilitation (*N* = 35)**	***F***	**$\eta_{p}^{2}$**
Restrained eating	23.30 (6.98)	26.62 (8.42)	24.29 (6.85)	23.00 (7.36)	1.79	0.04
Perceived dieting success	12.73 (3.89)	12.76 (3.80)	13.11 (3.24)	12.49 (3.28)	0.19	0.00
Eating self-regulation	15.76 (5.49)	18.22 (6.78)	18.11 (5.88)	17.86 (6.53)	1.31	0.03
BMI	22.17 (3.39)	22.29 (2.95)	22.31 (3.47)	22.45 (3.13)	0.05	0.00
Age	24.43 (4.61)	25.05 (6.54)	24.45 (4.23)	23.51 (4.97)	0.54	0.01
Hunger (before)	7.81 (2.86)	8.46 (2.80)	8.13 (3.07)	8.83 (3.18)	0.78	0.01
Hunger (after)	6.16 (2.39)	6.86 (2.15)	7.00 (2.90)	7.31 (2.99)	1.25	0.03
Hunger (change)	1.65 (2.12)	1.59 (2.25)	1.13 (1.70)	1.51 (2.16)	0.48	0.01
Mood (before)	273.16 (83.64)	295.14 (80.85)	296.82 (65.57)	302.00 (72.28)	1.03	0.02
Mood (after)	303.51 (72.24)	325.76 (72.45)	314.55 (59.74)	320.69 (63.04)	0.75	0.02
Mood (change)	30.35 (77.44)	30.62 (52.96)	17.74 (63.38)	18.69 (64.96)	0.44	0.01
Liking (before)	284.27 (110.13)	282.19 (129.24)	289.66 (119.50)	308.97 (89.01)	0.41	0.01
Liking (after)	305.22 (91.64)	294.35 (112.85)	296.34 (104.20)	326.09 (76.29)	0.79	0.02
Liking (change)	20.95 (65.59)	12.16 (85.09)	6.68 (76.45)	17.11 (47.40)	0.288	0.01
Sweets consumption	4.49 (2.27)	4.89 (2.32)	4.71 (2.03)	5.03 (1.93)	0.43	0.01
Cognitive demand	92.95_a_ (96.75)	71.57_b_ (71.41)	161.37_ab_ (111.91)	180.31_ab_ (123.79)	9.53^***^	0.17
Quality of imaginations	283.35 (94.02)	307.65 (88.69)	287.08 (75.27)	317.43 (81.75)	1.34	0.03

*** p < 0.001. Standard deviations appear in parentheses below means.

Means with differing subscripts within rows are significantly different at the p < 0.05 based on Fisher's LSD post hoc paired comparisons.

**Figure 1:**
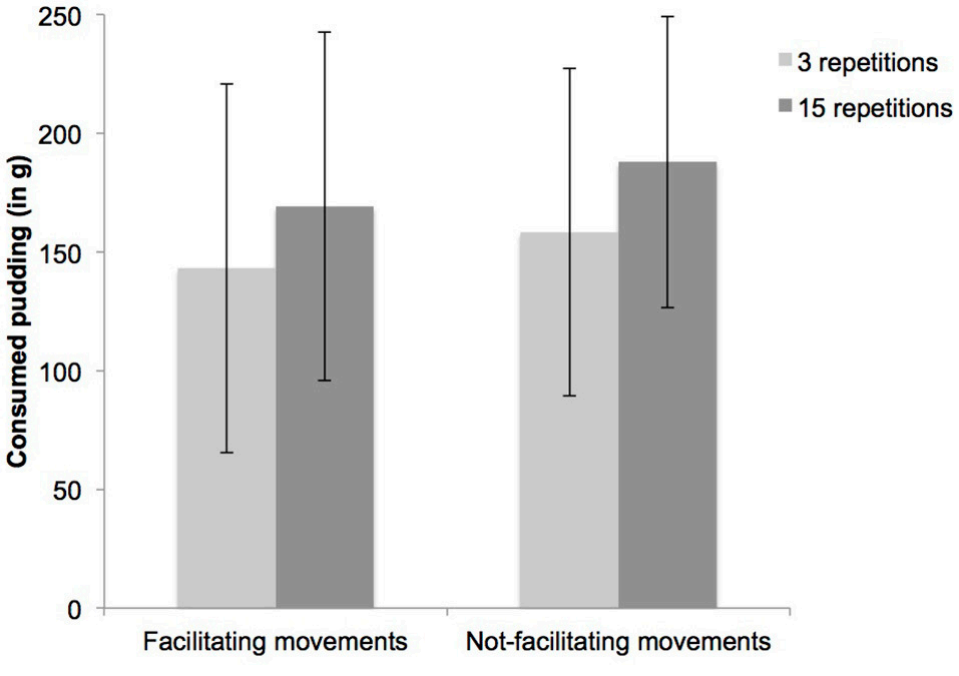
**Consumption of chocolate pudding as a function of 15 vs. 3 imagined consumption repetitions and facilitating vs. not-facilitating motor movements simulations**.

An ANOVA testing for the effects of imagined consumption repetitions and motor simulation did not reveal the expected interaction effect, *F*_(1, 143)_ = 0.02, *p* = 0.89. The analysis showed a main effect (see Figure 1) of imagined consumption repetitions, *F*_(1, 143)_ = 5.69, *p* = 0.02, $\eta_{p}^{2}$ = 0.04. Participants consumed more grams of pudding when they imagined the consumption 15 times (*M*_15_ = 178.20, *SD*_15_ = 68.08) than when they repeated it 3 times (*M*_3_ = 150.73, *SD*_3_ = 73.31). A subsequent between-subject ANOVA with cognitive demand as dependent variable revealed a main effect of imagined consumption repetitions, *F*_(1, 143)_ = 27.38, *p* < 0.001, $\eta_{p}^{2}$ = 0.16, where participants experienced greater cognitive demand when imagining consumption 15 times (*M*_15_ = 170.45, *SD*_15_ = 117.32) in comparison to imagining it 3 times (*M*_3_ = 82.26, *SD*_3_ = 85.13). An ANOVA with imaginations quality as dependent variable further showed a main effect of type of motor simulation, *F*_(1, 143)_ = 3.78, *p* = 0.05, $\eta_{p}^{2}$ = 0.03. Participants reported imagining consumption better when performing not-facilitating (*M*_*not-facilitation*_ = 312.40, *SD*_*not-facilitation*_ = 84.93) than when performing facilitating eating movements (*M*_*facilitation*_ = 285.24, *SD*_*facilitation*_ = 84.48). Change in hunger, mood or pudding liking did not reveal any effects of the experimental manipulations (all *p's* > 0.26). Controlling for these additional variables in an ANCOVA did not affect the obtained sensitization effect. Main effect of imagined consumption repetitions while controlled for cognitive demand [*F*_(1, 142)_ = 7.61, *p* = 0.01, $\eta_{p}^{2}$ = 0.05] and quality of imaginations [*F*_(1, 142)_ = 5.56, *p* = 0.02, $\eta_{p}^{2}$ = 0.04] remained stable (for further analysis of the additional variables, see Supplementary Datasheet 1).

To rule out potential effects of demand characteristics, we have also examined the distribution of participants expectations of how imagined consumption and motor simulation affects food intake. Most participants (49%) indicated that after imagining eating a particular food, a person would eat more of it, while 28.6% would expect a person to eat less after a consumption imagination—the actual habituation effect. No change in consumption behavior was expected by 13.6% and 8.8% reported a different opinion (e.g., “A person eats more after imagining eating a product one does not necessarily like.”). Further, 36.1% participants expected that simulation of facilitating motor movements would increase the amount of consumed food and vice versa for simulation of not-facilitating motor movements. The opposite trend representing our hypotheses, eating less after simulating facilitating and more after not-facilitating motor movements, was expected by 34.0% participants. No effect of either kind of motor movements on subsequent consumption was expected by 25.9% and 4.1% expressed a different opinion (e.g., “A person is more aware of her consumption.”). This distribution of participants' opinions does not seem to represent a prevalent case of a strongly perceived demand to behave a certain way.

## Discussion

Mental simulation of food consumption has been called upon as yet another prospective basis for developing interventions to effectively reduce food intake (Morewedge et al., 2010; Missbach et al., 2015). The main objective of this study was to investigate conditions under which the repeated imagined-consumption paradigm could be applied more efficiently. Therefore, we employed consumption-related motor simulation to strengthen the habituation-based consumption-reduction effect of the imagery paradigm at smaller numbers of imagined consumption repetitions to reduce the time demand. The results indicated that 15 repetitions of imagined consumption were not sufficient to produce habituation to a specific food (chocolate pudding). Indeed, 15 compared with 3 repetitions even led to an increase in the amount of food that participants consumed. The simulation of motor movements during imagination of food consumption did not enhance the hypothesized habituation process and neither reduced the obtained sensitization effect. Our results illustrate that the imagined consumption paradigm in a more compact form, utilizing fewer imagination repetitions and potential facilitator, does not lead to reduction in consumption and thus might represent a potential danger when applied in real-life interventions.

Exposure to appetitive food-related cues, visual, sensual and even imagined, often results in increased appetite (for a review see: Petrovich, 2013; Boswell and Kober, 2016), hunger (e.g., Jansen and van den Hout, 1991; Staiger et al., 2000) and desire to eat leading to increased food intake (e.g., Jansen et al., 2003, 2011; Rodríguez-Martín and Meule, 2015). Increase in responsiveness to a particular food is also reflected in sensitization, a phenomenon that precedes habituation (Epstein et al., 2009). Seeing a food for the first time leads to an initial increase in responsiveness to the food, increasing the foods' consumption, because it still represents novel information. Meanwhile, decrease in consumption due to habituation takes place when the food presentation is no longer surprising, because after repeated presentations the food information is well stored in memory and the responsiveness to the food drops (Epstein et al., 2009). Overall, our results imply that contrary to our hypotheses, the sensitization effect persisted and was more pronounced after 15 repetitions of imagined food consumption. One explanation for these results is provided by the characteristics of habituation processes. It is likely that environmental stimuli or activities (e.g., physical engagement) could affect the rate of decrease in responses and alleged thresholds, resulting in a more flexible duration of sensitization and habituation effects in (imagined) food consumption. Exposure, even repeated, to other stimuli during the habituation process can slow down the rate of decrease in responses (Epstein et al., 2009), resulting in a sensitization effect of longer duration. Particularly, the emerging of the sensitization effect in our study did not seem to be affected by experienced cognitive demand during the task or quality of the consumption imagination. This reflects the strength and generality of the sensitization effect.

A certain amount of caution needs to be however exercised when it comes to interpretation of our results in the context of habituation mechanisms. Procedural differences between our paradigm and previously applied paradigms to study habituation induced by consumption imagery could indeed offer further explanations of our results. For example, using dense, compact chocolate pudding as habituating stimulus could have increased participants eating motivation. Previous research tested the effects of imagined food consumption with food stimuli that were of discrete nature like pieces of M&Ms, cubes of cheese (Morewedge et al., 2010), or gummy beers and walnuts (Missbach et al., 2014). Results reported in the literature point out that larger bite sizes which can be greater with semisolid foods than small pieces of solid foods, can indeed increase food intake (Zijlstra et al., 2009). Similarly, it has been suggested that chewing a solid food can provide a satiety signal that is not apparent with swallowing a more liquid foods (Zijlstra et al., 2009). Because we did not include a replication condition with approximately 30 repetitions of imagined consumption, we cannot be certain that habituation would have occurred in our paradigm even under the standardly applied (Missbach et al., 2014) 30 repetition condition. Moreover, it appears that participants managed to successfully imagine fewer consumption repetitions than the instructed 15, raising the probability that no sufficient room was provided for the habituation, a process that requires time, to occur. These limitations should be addressed in future research.

Furthermore, it seems that simulation of facilitating in comparison to not facilitating motor movement did not accelerate enfolding of the habituation effect as assumed, but also did not impact the sensitization effect. We did not expect this result, because other studies have shown that eating-facilitating motor movements lead to increase in consumption. For example, Topolinski and Türk Pereira (2012) showed that food deprived individuals reported more hunger after they chewed a tasteless and calorie free chewing gum in comparison to those who kneaded a ball. Similarly, Topolinski and Boecker (2016) found that rehearsal of mouth movement's that resembled ingestion, signaling approach motivation (Topolinski et al., 2014) led to increased ratings of food palatability than rehearsing mouth movements associated with expectoration, signaling avoidance. Most relevantly, Förster (2003) showed that flexing an arm, representing approach movement, increased food consumption in contrast to avoidance associated movement of extending ones arm. However, there is an important difference of the procedures applied in our study and procedures applied in the mentioned studies. Here, eating-related movements are performed only during one consumption instance when congruence of motor simulation and consumption is allowed (Förster, 2003) or with a larger variety of foods (Topolinski and Boecker, 2016). In contrast, we tested motor movements in context of habituation, performed repeatedly, separately from consumption and associated with one specific food item only.

Taking into account that the procedure used in this study differed to the procedures of the studies mentioned above, we put forward an alternative explanation of the absenting moderating effects of motor movements. Repeated simulation of particular kind of physical movement for 15 times could have impeded habituation the same way in both, facilitating and not-facilitating, conditions. It can be speculated that rehearsing both types of motor movements in ritual-like fashion, defining behaviors repeated in fixed, episodic sequences (Schippers and van Lange, 2006), could have lead to increased personal involvement in the act of consumption. Consequently, we could have observed an increased food intake. In line with this argument is a recent research by Vohs et al. (2013, Experiment 4), which showed that ritual-like gestures increase personal involvement and lead to higher anticipated and actual enjoyment of subsequent consumption. Such anticipated enjoyment might drive the desire to eat (Stroebe et al., 2008). Nevertheless, it is important to note that we do not know whether increased repetition (e.g., 15 times) of ritual-like movements would linearly increase involvement, anticipated enjoyment and consumption itself.

Essentially, the current research clearly shows that the repeated imagined-consumption paradigm cannot be applied in interventions with a small number of repetitions and accompanied by other salient contextual factors, because it can actually backfire. Our results indicate that the technique of imagined consumption, using 15 imagery repetitions and motor simulation, can lead to the undesired consequence of increased food consumption. Moreover, this technique does not reduce food intake in situations where a person does not have enough cognitive resources (Missbach et al., 2014). The participants in our study also appeared to have successfully imagined the consumption fewer times than 15, despite the fact that each imagination repetition has been introduced separately in the audio instructions. Not complying with the explicitly instructed number of repetitions that might reach even 30, as to secure successful occurrence of the underlying habituation process, can illustrate the danger lying in real-life application of this technique. An additional side effect of this technique is that imagining consumption of one food, even with larger numbers of repetitions, can result in sensitization, an unwanted increase in the consumption of foods that are complementary to it. Huh et al. (2016), showed that imagining eating crackers 30 times resulted in increased consumption of cheese, a complementary food to crackers (see Experiment 4).

Hence, intervention programs should apply the repeated imagined consumption technique, and possibly other similar methods, only with caution. Other imagery-based techniques used to reduce food intake (e.g., mindfulness) might lead to similar consequences. Even though mindfulness techniques are not based on memory processes and do not require a large number of repetitions (Papies et al., 2015) their successful practice likely demands equal amounts of time and concentration. Additionally, these techniques—just like food consumption itself—might be subjected to the influence of external situational or environmental factors and cues that are often outside of people's awareness or acknowledgment (Vartanian et al., 2008; Stöckli et al., 2016). Both groups of aspects can potentially impair or at least flex the processes of mental imagery and additional mechanisms (e.g., habituation or mindful attention), resulting in undesired effects on consumption. Further research is needed to clarify when repeated thoughts of consumption lead to an increase or decrease in food intake and how different environmental contexts in which consumption takes place or other accompanying behaviors influence results. Continuing to increase efficiency of such techniques, future research might investigate other facilitators of the beneficiary effects of habituation on food intake reduction. Also, more insights into long-term effects of this paradigm are necessary for its practical application.

## Author contributions

BE and AF designed the study. BE conducted the study. BE, SH, and BM conducted the literature review and wrote the research summaries. SH and BE analyzed the data. SH wrote the first draft of the manuscript, and all authors contributed to and have approved the final manuscript. Authors had full access to the study data.

## Funding

This research did not receive any specific grant from funding agencies in the public, commercial, or non-profit sectors. The articles' publication was supported by the Open Access Publishing Fund of the University of Vienna, a source with no involvement in study design, data collection, analysis, data interpretation, or report writing and neither in publication submission decisions.

### Conflict of interest statement

The authors declare that the research was conducted in the absence of any commercial or financial relationships that could be construed as a potential conflict of interest.
