# Supplementary material for: Effects of Imagined Consumption and Simulated Eating Movements on Food Intake: Thoughts about Food Are Not Always of Advantage
Source: Front Psychol. 2016 Oct 28;7:1691. doi: 10.3389/fpsyg.2016.01691 (PMC5084053; doi:10.3389/fpsyg.2016.01691)
Supplement: Supplementary file 1 [file DataSheet1.DOCX]

Supplementary Material

**Effects of Imagined Consumption and Simulated Eating Movements on Food Intake: Thoughts about Food are not Always of Advantage.**

Simona Haasova^*^, Botond Elekes, Benjamin Missbach, Arnd Florack

*** Correspondence:** Simona Haasova: simona.haasova@univie.ac.at

# The present study

## Participants

Participants were recruited via social media and flyers posted on University campus. First, potential participants filled out a short online questionnaire addressing the study’s inclusion criteria and measuring scales of interest independently to avoid any influence of the experimental procedure on the measurements or vice versa. Five hundred thirty-six participants completed the online questionnaire and responded to questions about their age, gender, diet, and food allergies, and reported on their restrained eating, subjective dietary success and additional questions on eating self-regulation. We applied three inclusion criteria: 1. age between 18 and 54 years, because higher age has substantial impact on imagery processes (Dror & Kosslyn, 1994), 2. absence of soya intolerance, because the food stimulus used in the study included soya, and 3. absence of current dietary restraints. Based on the inclusion criteria, we invited 334 participants to take part in the experiment in laboratory settings. One hundred fifty-nine participants, predominantly students, volunteered to participate in the laboratory experiment in exchange for course credits or the opportunity to win 5 EUR in a lottery. We excluded 12 participants from the data analysis due to either an initially reported dislike towards eating the food - chocolate pudding - in the study or no response on the “liking” measure (8), due to expressed aversion to eat the food during the “taste test” task (2), due to incomplete responses on the online questionnaires containing measures of control variables (1) and because one participant explained to eat less in the “taste test” task than desired, being afraid to experience allergic reaction. The final sample consisted of 147 participants (115 female) with a mean age of 24.37 (*SD* = 5.14).

## Design and procedure

We asked participants to refrain from eating and drinking calorie-dense soft drinks 2 hours prior to the experiment. The participants believed they are taking part in a taste test of a product, specifically, a taste test of a chocolate pudding - we chose the Silky Smooth Chocolate Dessert by Alpro. Participants were tested individually, while all instructions were provided on a computer screen or as audio files played through a headset. The duration of one experimental session was approx. 30 minutes, participants were tested individually.

After arriving at the laboratory, participants signed the informed consent and were then randomly assigned to four conditions, representing the 2 (imagined consumption repetitions: 15 vs. 3) x 2 (motor simulation: facilitating vs. not-facilitating) between-subjects design. Next, participants answered questions about their age, gender, height and weight (for BMI calculation), and indicated their current hunger, mood, and liking of chocolate pudding as well as their weekly frequency of sweets consumption. The alleged taste test was preceded by the mental imagery task and motor movements simulation task. Participants repeatedly imagined consuming the chocolate pudding either 15 or 3 consecutive times and concurrently performed either consumption facilitating or not-facilitating movements. Subsequently, participants reported on subjectively perceived cognitive demand during the joint task, subjective quality of imagined consumptions, and manipulation-check questions: liking of the voice from the audio instructions, experienced disturbance during the task and overall instructions compliance. Following, a taste test of the previously imagined chocolate pudding took place, during which participants could eat the chocolate pudding at libitum. Afterwards, participants again reported their feelings of hunger, mood and liking of chocolate pudding. In addition, participants also indicated their expectations on how imagining consumption and motor simulation influences subsequent pudding consumption. Participants then received their payment (in the case of lottery winnings) or were granted course credit for their participation, were thanked and debriefed.

### Imagined consumption and motor movements simulation task.

At the beginning of the joint mental imagery task, participants put on earphones and were instructed step by step by a calm, female voice to imagine how they would eat the chocolate pudding, receiving instructions with each of the ascribed repetitions, holding so the time for each imagination constant at cca 40 s. We instructed them to imagine how they put a spoon into the chocolate pudding, move the spoon towards their mouth, smell the scent of the pudding, taste and swallow it, emphasizing so the visual, olfactory and haptic properties of the food. We instructed participants to repeat imagining the consumption 3 or 15 times. Simultaneously, along with each imagination repetition, participants were instructed to actually move their hand holding an imagined spoon to the mouth (consumption facilitating movement), or to keep their hands still and hold their tongue behind their teeth (consumption not-facilitating movements), respective of their assigned condition. We emphasized to the participants that the goal was to create imaginations as vivid and clear as possible. Further, we asked participants to draw a dash on a sheet of paper for every repetition when they have successfully managed to imagine consuming the chocolate pudding.

### Taste test.

After the mental imagery task, participants engaged in an 8-minutes long taste test of the previously imagined chocolate pudding. We instructed the participants to uncover a black box standing next to the screen to find a metallic spoon and a glass bowl containing 250 g of the chocolate pudding (representing approx. 2 packages), from which they could eat ad libitum. We measured the weight of the bowl before and after every appointment, otherwise not being present during participants’ consumption. Subsequently, following the taste test scenario, participants answered evaluative questions about the chocolate pudding (e.g., “What did you like best about the chocolate pudding?”, “How did you like the taste?” etc.). These responses were not relevant to our research question and were therefore not addressed in the analysis further.

## Measures

### Restrained eating (RE).

We assessed RE with the Restrained eating subscale that consists of 10 items on a 5-point rating scale ranging from 1 (*never*) to 5 (*very often*), from the Dutch Eating Behavior Questionnaire, using its established German version (Grunert, 1989; originally published in Dutch: Van Strien, Freijters, Bergers, & Defares, 1986). The scale demonstrated very good internal consistency, α = .88.

### Perceived dieting success (PDS).

To measure PDS, participants completed the Perceived Self-Regulatory Success in Dieting Scale (Meule, Papies, & Kübler, 2012), consisting of three items on a 7-point rating scale ranging from 1 (*not at all difficult/ not at all good*) to 7 (*very difficult/ very good*). Participants were asked how successful they were in keeping their weight, in losing extra weight and in staying in shape (last item reverse-coded). The scale demonstrated satisfactory internal consistency, α = .73.

### Eating self-regulation (ES).

The Advanced Self Regulatory Scale (“Erweiterte Selbstregulation Skala”) was developed in our laboratory to measure ES. The scale consists of four items on a 7-point rating scale ranging from 1 (*I don’t agree at all*) to 7 (*I fully agree*): 1.“When I am full, I stop eating.”; 2.“It is easy for me to stop eating, when I feel no more hunger.”; 3. “I often eat further, even though my stomach feels full.”: 4. “I often keep eating, even though I am not hungry anymore.” (Last two items reverse-coded). The scale demonstrated high internal consistency, α =.90.

### Hunger.

We measured participants’ hunger before and after the mental imagery task with the Hunger subscale of the German version of the Food Cravings Questionnaire-State (Meule, Lutz, Vögele, & Kübler, 2012). The subscale consists of three items (e.g., “I am hungry”) on a 7-point rating scale ranging from 1 (*I don’t agree at all*) to 7 (*I fully agree*). The scales internal consistency was satisfactory at both time points (before: α = .83; after: α = .78).

### Pudding liking.

Participants’ liking of the chocolate pudding was also assessed before and after the mental imagery task with the item: “How much do you like chocolate pudding?” using a 400 point visual analog scale (VAS) ranging from 1 (*not at all*) to 400 (*very much*).

### Mood.

Similarly, we measured participants’ mood before and after the mental imagery task with the item: “How do you feel at this moment?” with a 400 point VAS ranging from 1 (*sad*) to 400 (*happy*).

### Body Mass Index (BMI).

Collecting data about participants’ weight and height, we calculated BMI as weight (in kilogram) divided by the squared size of height (in meters).

### Sweets consumption (SC).

In order to assess individual frequency of sugary meals and snacks consumption (e.g., chocolate bars, candy, desserts), we asked participants “How often do you consume sweet meals, desserts, or generally sweets during the week on average?” with a 8-point rating scale ranging from 0 (*never*), 1 (*one day a week*) to 7 (*everyday*).

### Cognitive demand (CD).

To asses the amount of perceived cognitive demand during the mental imagery task, we asked our participants to indicate “How demanding did you perceive the task altogether?” with a 400 point VAS ranging from 1 (*not at all demanding*) to 400 (*very demanding*).

### Quality of imaginations (QI).

Measuring the quality of the performed consumption imaginations, we asked our participants to indicate “How good could you imagine consuming the food?” on a 400 point VAS ranging from 1 (*very badly*) to 400 (*very good*).

### Manipulation check.

We evaluated whether participants complied with the task instructions with the questions: “Did you imagine the pudding consumption according to the instructions?” on a 2-point scale, 1 (*yes) and 2* (*no*) and “How many times did you successfully manage to imagine consuming the chocolate pudding?”, where participants reported their noted number. Further, we asked whether they perceived any difficulties during the task (“Did you experience any problems during the task performance due to disturbances?”; binary response: yes, no) and how pleasantly they judged the voice delivering audio instructions (“How did you find the female voice from the audio recording?”; 4-point rating scale from 1 (*very pleasant*) to 4 (*very unpleasant*).

# Results

## Preliminary analysis

Table 1 depicts descriptive and inferential statistics of the study´s variables between experimental conditions. The four experimental conditions did not differ in participants’ RE, PDS or ES, neither in age or gender (χ^^ (3, *N* = 147) = 6.18, *p* = .10). Participants reported similar feelings of hunger, mood and chocolate pudding liking at the start of the study in all experimental conditions. During the manipulation check, all participants indicated they have fully complied with the given task instructions and managed to successfully imagine the consumption of the chocolate pudding more times in 15 (M_15_ = 10.49, SD_15_ = 3.83) vs. 3 repetition conditions (M_3_ = 2.58, SD_3_ = .76; *F*(1, 145) = 303.73 , *p* < .001, η_p_^2^ = .68) and perceived the instructing female voice as rather pleasant (*M* = 2.85; *SD* = .63). Sixteen participants reported they have experienced some disturbances during the experimental task (e.g., occasional external sounds). Excluding these participants from statistical analysis did not change the findings (main effect of imagined consumption repetitions: *F*(1, 127) = 4.97, *p* = .03, η_p_^2^ ^ ^= .04), thus we use the full sample in further analysis.

Table 2 depicts correlations between the study´s variables. Corresponding with previous research ([Meule et al., 2012](#_ENREF_7)), PDS was negatively correlated with BMI, and also RE.

## Main analysis

A between-subjects ANOVA with number of imagined consumption repetitions and type of motor simulation as independent variables and amount of consumed pudding as the dependent variable revealed a main effect of imagined consumption repetitions, *F*(1, 143) = 5.69, *p* = .02, η_p_^2^ = .04, where participants consumed more pudding when they imagined the consumption 15 times (*M_15_* = 178.20, *SD_15_* = 68.08) than when they repeated it 3 times (*M_3_* = 150.73, *SD_3_* = 73.31). Contrary to the expected habituation effect and interaction between number of repetitions and type of motor movements, we obtained a sensitization effect, characteristic for increase in consumption occurring shortly before its decrease due to habituation.

## Additional analysis

An ANOVA with perceived cognitive demand as dependent variable and both experimental factors as independent variables showed a main effect of imagined consumption repetitions, *F*(1, 143) = 27.38, *p* < .001, η_p_^2^ = .16). Participants who performed 15 consumption imaginations (*M_15_* = 170.45 *SD_15_* = 117.32) experienced the task as more demanding than participants who performed only 3 repetitions (*M_3_* = 82.26, *SD_3_* = 85.13). However, the perceived CD did not correlate with the amount of subsequently consumed pudding (*r(*145) = -.03, *p* = .74) and therefore we conclude that increased CD does not mediate the relationship between higher number of imagination repetitions and increased consumption, indicating that the sensitization effect is not an artifact of participants’ exhaustion or tiredness.

A an ANOVA with IQ as dependent variable further showed a main effect of type of motor simulation, *F*(1, 143) = 3.78, *p* = .05, η_p_^2^ = .03. Participants reported imagining consumption better when performing not-facilitating (*M_not-facilitation_* = 312.40, *SD_not-facilitation_* = 84.93) than when performing facilitating eating movements (*M_facilitation_* = 285.24, *SD_facilitation_* = 84.48). Again, the IQ did not correlate with the amount of subsequently consumed pudding (*r(*145) = .07, *p* = .43).

In order to assess the changes of hunger, mood or pudding liking in the experimental procedure and their potential relations with the sensitization effect, we applied repeated measures ANOVA with hunger (mood, liking) measurement from before and after the experimental procedure as dependent variable and number of imagined consumption repetitions and type of motor simulation as a between subject independent factors. The analyses revealed main effects of time of measurement: after the experimental procedure, participants felt less hungry (*F*(1, 143) = 74.58, *p* < .001, η_p_^2^ = .34; *M_before_* = 8.30, *SD_before_* = 2.97; *M_after_* = 6.83, *SD_after_* = 2.63), more happy (*F*(1, 143) = 20.45, *p* < .001, η_p_^2^ = .13; *M_before_* = 291.67, *SD_before_* = 75.93; *M_after_* = 316.05, *SD_after_* = 66.94) and liked the chocolate pudding better (*F*(1, 143) = 6.00, *p* = .02, η_p_^2^ = .04; (*M_before_* = 291.02, *SD_before_* = 112.62; *M_after_* = 305.16, *SD_after_* = 97.33). We found no effects of imagination repetitions or type of motor simulation (all *p´s* > .26). These findings further illustrate that the observed sensitization effect did not occur due to larger motivation to reduce hunger, to increase mood or increased liking of the pudding within participants who imagined consuming the chocolate pudding 15 times.

Participants´ age correlated with the pudding consumption (*r(*145) = -.20, *p* = .02), but gender did not (*r(*145) = -.09, *p* = .27).

None of the additionally assessed variables affected the obtained sensitization effect: initial hunger (*F*(1, 142) = 5.24, *p* = .02, η_p_^2^ = .04); initial mood (*F*(1, 142) = 5.56, *p* = .02, η_p_^2^ = .04); initial liking of the chocolate pudding (*F*(1, 142) = 4.98, *p* = .03, η_p_^2^ = .03); SC (*F*(1, 142) = 5.84, *p* = .02, η_p_^2^ = .04); BMI scores (*F*(1, 142) = 5.85, *p* = .02, η_p_^2^ = .04); RE (*F*(1, 142) = 4.92, *p* = .03, η_p_^2^ = .03); PSD (*F*(1, 142) = 6.01, *p* = .02, η_p_^2^ = .04); ES (*F*(1, 142) = 6.03 *p* = .02, η_p_^2^ = .04); CD (*F*(1, 142) = 7.61, *p* = .01, η_p_^2^ = .05); QI (*F*(1, 142) = 5.56, *p* = .02, η_p_^2^ = .04).

# References

Dror, I. E., & Kosslyn, S. M. (1994). Mental imagery and aging. *Psychology and Aging, 9*(1), 90-102.

Grunert, S. C. (1989). Ein Inventar zur Erfassung von Selbstaussagen zum Ernährungsverhalten [An inventory for the assessment of self-statements about eating habits]. *Diagnostica, 35*(2), 167-179.

Meule, A., Lutz, A., Vögele, C., & Kübler, A. (2012). Food cravings discriminate differentially between successful and unsuccessful dieters and non-dieters. Validation of the Food Cravings Questionnaires in German. *Appetite, 58*(1), 88-97.

Meule, A., Papies, E. K., & Kübler, A. (2012). Differentiating between successful and unsuccessful dieters. Validity and reliability of the Perceived Self-Regulatory Success in Dieting Scale. *Appetite, 58*(3), 822-826.

Van Strien, T., Frijters, J. E., Van Staveren, W. A., Defares, P. B., & Deurenberg, P. (1986). The predictive validity of the Dutch restrained eating scale. *International Journal of Eating Disorders, 5*(4), 747-755.

Table 1

*Descriptive (means and standard deviations) and inferential statistics of study´s variables between the four experimental conditions.*

| Study´s Variables | Experimental conditions | | | |  |  |
| --- | --- | --- | --- | --- | --- | --- |
|  | 3 repetitions  motor facilitation (N = 37) | 3 repetitions  motor not-facilitation (N = 37) | 15 repetitions  motor facilitation  (N = 38) | 15 repetitions  motor not- facilitation  (N = 35) | *F* | η_p_^2^ |
| RE | 23.30 | 26.62 | 24.29 | 23.00 | 1.79 | .04 |
|  | (6.98) | (8.42) | (6.85) | (7.36) |  |  |
| PDS | 12.73 | 12.76 | 13.11 | 12.49 | .19 | .00 |
|  | (3.89) | (3.80) | (3.24) | (3.28) |  |  |
| ES | 15.76 | 18.22 | 18.11 | 17.86 | 1.31 | .03 |
|  | (5.49) | (6.78) | (5.88) | (6.53) |  |  |
| BMI | 22.17 | 22.29 | 22.31 | 22.45 | .05 | .00 |
|  | (3.39) | (2.95) | (3.47) | (3.13) |  |  |
| Age | 24.43 | 25.05 | 24.45 | 23.51 | .54 | .01 |
|  | (4.61) | (6.54) | (4.23) | (4.97) |  |  |
| Hunger  (before) | 7.81  (2.86) | 8.46  (2.80) | 8.13  (3.07) | 8.83  (3.18) | .78 | .01 |
| Hunger  (after) | 6.16  (2.39) | 6.86  (2.15) | 7.00  (2.90) | 7.31  (2.99) | 1.25 | .03 |
| Hunger (change) | 1.65  (2.12) | 1.59  (2.25) | 1.13  (1.70) | 1.51  (2.16) | .48 | .01 |
| Mood  (before) | 273.16  (83.64) | 295.14  (80.85) | 296.82  (65.57) | 302.00  (72.28) | 1.03 | .02 |
| Mood  (after) | 303.51  (72.24) | 325.76  (72.45) | 314.55  (59.74) | 320.69  (63.04) | .75 | .02 |
| Mood  (change) | 30.35  (77.44) | 30.62  (52.96) | 17.74  (63.38) | 18.69  (64.96) | .44 | .01 |
| Liking  (before) | 284.27  (110.13) | 282.19  (129.24) | 289.66  (119.50) | 308.97  (89.01) | .41 | .01 |
| Liking  (after) | 305.22  (91.64) | 294.35  (112.85) | 296.34  (104.20) | 326.09  (76.29) | .79 | .02 |
| Liking  (change) | 20.95  (65.59) | 12.16  (85.09) | 6.68  (76.45) | 17.11  (47.40) | .288 | .01 |
| SC | 4.49  (2.27) | 4.89  (2.32) | 4.71  (2.03) | 5.03  (1.93) | .43 | .01 |
| CD | 92.95_a_ | 71.57_b_ | 161.37_ab_ | 180.31_ab_ | 9.53^***^ | .17 |
|  | (96.75) | (71.41) | (111.91) | (123.79) |  |  |
| QI | 283.35 | 307.65 | 287.08 | 317.43 | 1.34 | .03 |
|  | (94.02) | (88.69) | (75.27) | (81.75) |  |  |
|  |  |  |  |  |  |  |

*Note*. * = *p* < .05, ** = *p* < .01, *** = *p* < .001. Standard deviations appear in parentheses below means. Variables shortcut names in the table stand for: RE = restrained eating; PSD = perceived dietary success, ES = eating self-regulation, SC = frequency of sweets consumption, CS = cognitive demand, QI = quality of imaginations. Means with differing subscripts within rows are significantly different at the *p* < .05 based on Fisher’s LSD post hoc paired comparisons.

Table 2

*Pearson´s correlations among study´s variables.*

|  | Study´s Variables | M | SD | 1 | 2 | 3 | 4 | 5 | 6 | 7 | 8 | 9 | 10 | 11 | 12 | 13 | 14 | 15 |
| --- | --- | --- | --- | --- | --- | --- | --- | --- | --- | --- | --- | --- | --- | --- | --- | --- | --- | --- |
| 1. | RE | 24.32 | 7.48 | 1 |  |  |  |  |  |  |  |  |  |  |  |  |  |  |
| 2. | PSD | 12.78 | 3.53 | - .28** | 1 |  |  |  |  |  |  |  |  |  |  |  |  |  |
| 3. | ES | 17.48 | 6.21 | -.02 | .11 | 1 |  |  |  |  |  |  |  |  |  |  |  |  |
| 4. | BMI | 22.32 | 3.21 | .08 | -.43** | .03 | 1 |  |  |  |  |  |  |  |  |  |  |  |
| 5. | Age | 24.37 | 5.14 | .02 | .14 | .20* | .17* | 1 |  |  |  |  |  |  |  |  |  |  |
| 6. | Hunger (before) | 8.30 | 2.97 | -.02 | .10 | -.04 | -.09 | -.04 | 1 |  |  |  |  |  |  |  |  |  |
| 7. | Hunger (after) | 6.83 | 2.63 | -.07 | .12 | -.03 | -.09 | .06 | .74** | 1 |  |  |  |  |  |  |  |  |
| 8. | Mood  (before) | 291.67 | 75.93 | .04 | .01 | .05 | .05 | -.04 | -.11 | .01 | 1 |  |  |  |  |  |  |  |
| 9. | Mood  (after) | 316.05 | 66.94 | .07 | .08 | .01 | -.01 | -.15 | -.06 | -.09 | .59** | 1 |  |  |  |  |  |  |
| 10. | Liking  (before) | 291.02 | 112.62 | .06 | -.02 | -.06 | .08 | -.11 | .12 | .14 | .12 | .20* | 1 |  |  |  |  |  |
| 11. | Liking  (after) | 305.16 | 97.33 | .06 | .05 | -.10 | .05 | -.07 | .08 | .01 | .15 | .39** | .79** | 1 |  |  |  |  |
| 12. | SC | 4.78 | 2.13 | -.03 | -.08 | -.09 | -.03 | .01 | -.07 | .00 | .-.12 | .05 | .11 | .15 | 1 |  |  |  |
| 13. | CD | 126.05 | 111.21 | -.05 | -.02 | -.01 | -.11 | .00 | .01 | .09 | -.06 | -.26** | -.03 | -.06 | .07 | 1 |  |  |
| 14. | QI | 298.54 | 85.50 | -.11 | -.01 | -.04 | .01 | .03 | .00 | -.14 | .01 | .08 | .05 | .04 | .01 | -.18* | 1 |  |
| 15. | Consumption | 164.37 | 71.85 | -.18* | .27** | -.05 | -.10 | -.20* | .18* | .01 | .03 | .27** | .25** | .40** | .-05 | -.03 | .07 | 1 |

*Note*. * = *p* < .05, ** = *p* < .01, *** = *p* < .001. *N* = 147 for all analysis.
